# Mechanical Work and Long-Distance Performance Prediction: the Influence of Allometric Scaling

**DOI:** 10.2478/hukin-2013-0047

**Published:** 2013-10-08

**Authors:** Marcus Peikriszwili Tartaruga, Jeanick Brisswalter, Carlos Bolli Mota, Cristine Lima Alberton, Natalia Andrea Gomeñuka, Leonardo Alexandre Peyré-Tartaruga

**Affiliations:** 1 Midwest State University of Paraná, LABIER, Guarapuava, Brazil.; 2 Federal University of Rio Grande do Sul, LAPEX, Porto Alegre, Brazil.; 3 University of Nice Sophia Antipolis, LAMHESS, Nice, France.; 4 Federal University of Santa Maria, Laboratory of Biomechanics, Santa Maria, Brazil.; 5 Federal University of Pelotas, School of Physical Education, Pelotas, Brazil.

**Keywords:** allometry, body size, cost of running, human locomotion, mechanical efficiency, running economy

## Abstract

The purpose of this study was to examine the effect of allometric scaling on the relationship between mechanical work and long-distance running performance in recreational runners. Fourteen recreational long-distance runners (male, mean ± SD - age: 29 ± 7 years; body mass: 70.0 ± 10.2 kg; body height: 1.71 ± 0.07 m; maximal oxygen uptake: VO_2max_ 52.0 ± 4.9 ml·kg^−1^·min^−1^) performed two tests: a continuous incremental test to volitional exhaustion in order to determine VO_2max_, and a 6-minute running submaximal test at 3.1 m·s^−1^, during which segments in the sagittal plane were recorded using a digital camera and the internal (W_int_), external (W_ext_) and total (W_tot_) mechanic work, in J·kg^−1^·m^−1^, was subsequently calculated. The results indicated a significant correlation between mechanical work and performance, however, the strongest correlations were observed when allometric exponents were used (respectively for W_int_, W_ext_ and W_tot_; non allometric vs. allometric scaling defined by literature (0.75) or determined mathematically (0.49): r = 0.38 vs. r = 0.44 and r = 0.50; r = 0.80 vs. r = 0.83 and r = 0.82; r = 0.70 vs. r = 0.77 and r = 0.78). These results indicate that mechanical work could be used as a predictor of recreational long-distance performance and an allometric model may improve this prediction.

## Introduction

An increase in participation in different forms of endurance running events has led to a need to better understand the factors that determine middle- and long-distance runners performance. Classically, the parameters of maximal oxygen uptake (VO_2max_), running efficiency and anaerobic threshold have been used to predict this performance (Saunders et al., 2004). The energy consumption in locomotion has been extensively studied and the relationship with the mechanical work investigated (Minetti et al., 1994). A number of biomechanical factors influences on running efficiency for example the ability of muscles to store and release elastic energy by increasing the stiffness of muscles, and more efficient mechanics leading to less energy wasted on braking forces and excessive vertical oscillation (Saunders et al., 2004).

Classically the mechanics of human locomotion have been analyzed from the mechanical work performed (Cavagna and Kaneko, 1977). This total mechanical work of locomotion (W_tot_) is traditionally considered as the sum of the two separate entities: external mechanical work (W_ext_) and internal mechanical work (W_int_) (Saibene and Minetti, 2003). W_ext_ represents the work necessary to lift and accelerate the body center of mass within the environment; and it has been investigated in many different conditions and populations (Saibene and Minetti, 2003). Calculating W_ext_ involves measuring the gravitational potential energy (PE) and the kinetic energy (KE) of the body center of mass, before calculating the total energy (TE = PE + KE) over time (Saibene and Minetti, 2003). This goal can be achieved both by using dynamometric (direct dynamics) and motion analysis (inverse dynamics) techniques (Cavagna, 2010).

The reciprocal movements of body segments that do not affect the trajectory of the body center of mass are, to a large extent, brought about by forces internal to the body and, consequently, work associated with energy changes relative to the body center of mass correspond to W_int_ (Willems et al., 1995). Therefore, W_int_ represents the work necessary to accelerate the limbs reciprocally with respect to the body center of mass during human locomotion and it is computed using both segment movements and anthropometric parameters (Cavagna, 2010).

Some authors have also investigated how the athlete’s anthropometric parameters and particularly body size can affect performance (Brisswalter et al., 1996; Ingham et al., 2008). The effect of body size for example, can be observed for Kenyan and Ethiopian runners who often dominate middle- and long-distance events in athletics. For these subjects running is systematically more economical because they generally have a smaller body size and thinner lower limbs than other runners (Foster and Lucia, 2007). Within this framework, some researchers have suggested the use of allometric scaling for efficiency assessment in order to take into account the effect of anthropometric characteristics on metabolic measures, in particular on running efficiency (Darveau et al., 2002; Nevill et al., 2004; Tartaruga et al., 2010). Running efficiency is defined as the steady-state submaximal oxygen uptake at given velocities (running economy – *RE*) (Saunders et al., 2004) or from energy expenditure per-unit distance (energy cost of running – *Cr*) (Minetti et al., 1994). These authors tried to demonstrate that an indiscriminate use of the unit ml·kg^−1^·min^−1^ is inappropriate for the purposes of comparing running efficiency between subjects with different body characteristics (Bergh et al., 1991) or performance level (Markovic et al., 2007), and suggested the use of kg^0.75^ or kg*^b^*, were *b* is the allometric or specific allometric exponent.

Within this framework, it has been well demonstrated that running efficiency depends to a large extent on locomotion mechanics (Saunders et al., 2004), therefore, to the best of our knowledge, no existing study has examined the influence of allometric scaling on the relationship between mechanical work (W_int_, W_ext_ and W_tot_) and the performance in long-distance runners. Thus, the purpose of this study was to examine the effect of allometric scaling on the relationships between mechanical work and the performance of recreational long-distance runners. This experiment was designed to test the hypothesis that the allometric model may improve predictions of the performance for recreational long-distance runners.

## Material and Methods

### Participants

Fourteen recreational long-distance runners (male, mean ± *SD* - age: 29 ± 7 years; body mass: 70.0 ± 10.2 kg; body height: 1.71 ± 0.07 m; lower limb length: 0.80 ± 0.03 m) participated in this study. Subjects were free of any musculoskeletal, bone and joint, or cardiac and pulmonary diseases and were not taking any medications. Calculation of the sample “*n*” was carried out using the PEPI program (Version 4.0) with a power of 90%.

Prior to participation, subjects were carefully informed of the design of the study, especially the possible risks and discomforts related to the procedures. Subjects then gave their written, informed consent. The institution’s Research Ethics Committee approved the present study according to the Declaration of Helsinki.

### Experimental procedures

**Figure 1:**
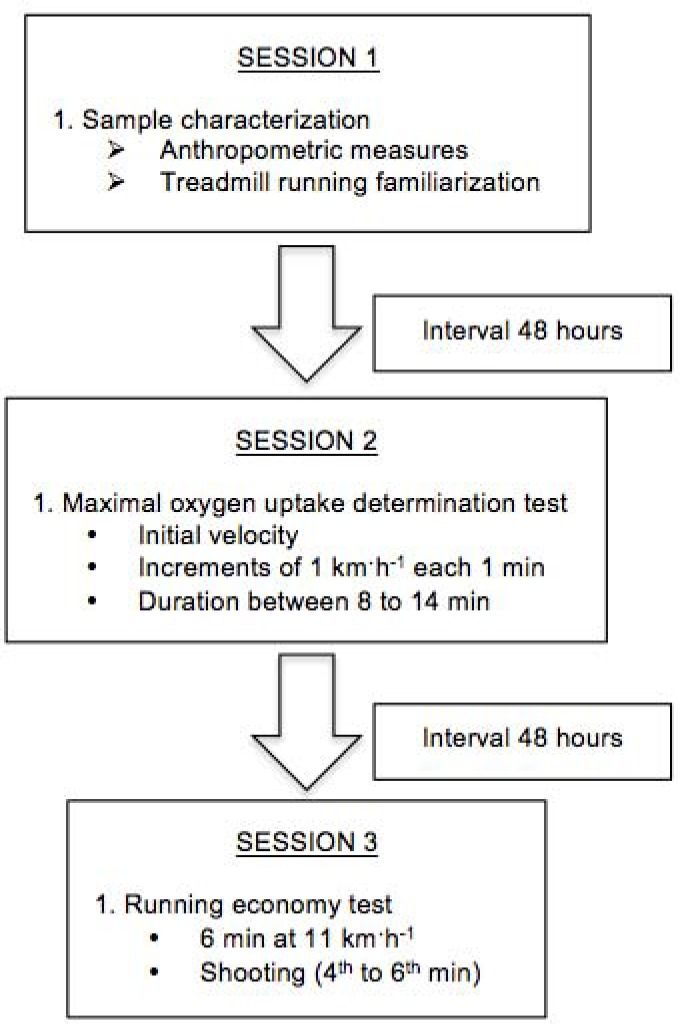
Experimental laboratory sessions

Each subject took part in three experimental laboratory sessions (sample characterization, incremental treadmill running protocol and running economy test) (Figure 1), with an interval of 48 h between each session. The laboratory ambient temperature (25°C) and relative humidity (53%) were controlled according to ISO-8573-1 (International Standards). Some restrictions were imposed on the subjects: no food 3–4 h before the tests and any stimulants or intense physical activity 12 h before each evaluation.

### Sample characterization

Body mass and height were measured using an analog medical scale (resolution of 0.1kg) and a stadiometer (FILIZOLA; Sao Paulo, Brazil) while the runners wore minimal clothing. Two experienced anthropometrists measured each subject. During the sample characterization session, all subjects participated in a familiarization exercise during which they were introduced to the process of treadmill running. In addition, all details of the care that would need to be taken while performing the exercise were explained in full.

### Incremental treadmill running protocol

After a brief warm-up and 10 min rest (5 min sitting and 5 min standing), subjects followed a progressive protocol on a treadmill (model 250 RT, MOVEMENT; Pompéia, Brazil) with an initial velocity of 2.8 m·s^−1^, in which speed was increased by 0.28 m·s^−1^ at 1 min intervals and treadmill incline was fixed at 1%. Load increments were calculated to reach VO_2max_ between 8 and 14 min. The VO_2max_ attainment criteria described by Howley et al. (1995) were adopted. Respiratory parameters were continually recorded using a mixing-box-type portable gas analyzer (model Aerosport KB1-C, INBRAMED; Ann Arbor, MI, USA). The gas analyzer was calibrated prior to each session.

### Running economy test

**Figure 2:**
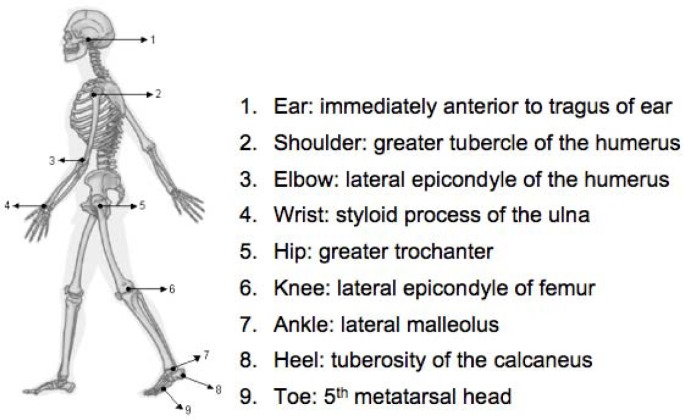
Localization of the retro-reflective markers

The running economy test consisted of a 6 min run at 3.1 m·s^−1^ (10% below the velocity at the anaerobic threshold) on a treadmill. Nine reflexives hemispherical spot markers were placed on the left sagittal plane to identify the subject’s segments of interest, as described by Willems et al. (1995) (Figure 2). The moving body segments were recorded during the last 20 s of the run using a digital camera (240 Hz; model EXFH25, CASIO; Tokyo, Japan).

### Performance

Performance was assessed using the fastest 10000 m run performed at the latest date in the month following laboratory testing.

### Allometric scaling

The effect of allometric scaling on the relationship between mechanical work and running performance was investigated using an allometric exponent (*b*) of 0.75 proposed by Bergh et al. (1991) and determined according to following general allometric equation:

(1) $$y=ax^{b},$$

where *y* is absolute VO_2max_, *x* is body mass and *a* is a constant characteristic for the organism, which is called the allometric coefficient (Jensen et al., 2001). For determination of specific *b*, the exponential function was transformed into a linear function:

(2) log y=b⋅log x+log a,

where *b* is the slope of the linear regression line on a double logarithmic plot. When *b* is 1 (Equation 1) the relationship is isometric and when it is higher or lower than 1, the relationship is allometric.

### Mechanical work

The total positive mechanical work (W_tot_) needed to sustain locomotion comprises positive internal mechanical work (W_int_), which is the work done to accelerate the body segments relative to body centre of mass, and positive external mechanical work (W_ext_), which is the work done to lift and accelerate the body centre of mass relative to the environment (Cavagna and Kaneko, 1977). This was computed from mechanical energies of the body segments determined from segment kinematics (Cavagna, 2010).

The determination of W_int_ requires computation of the instantaneous kinetic energy (KE) of each segment relative to the body centre of mass (KE_r_). This was performed as follows:

(3) $${KE}_{\text{r}}={½{m\nu }^{2}}_{\text{ap},\text{r}}+{½{m\nu }^{2}}_{\text{v},\text{r}}+½mK^{2}\omega^{2}$$

where *m* is the mass of the segment, *ν*_ap,r_ and *ν*_v,r_ are the antero-posterior and vertical components of the linear velocities of each segment relative to body centre of mass velocities, *K* is segment radius of gyration, and *ω* is angular velocity of the each segment. Instantaneous KEr of each segment within the same limb was then summed to give the kinetic energies of the upper and lower limbs, and the head-trunk. Internal work of each limb and of the head-trunk is then obtained by summing the positive increments of the KE curve of each limb and of the head-trunk segment separately over an integral number of strides. Overall W_int_ is then obtained as the sum of the internal work of each limb and the head-trunk segment. This computational scheme assumes energy transfers take place between segments of the same limb but not between limbs or between the trunk and limb.

The determination of W_ext_ requires computation of the instantaneous total mechanical energy of the body centre of mass (E_CM_).

This was performed as follows:

(4) $$E_{\text{CM}}=\text{Mgh}+{½{MV}^{2}}_{\text{ap}}+{½{MV}^{2}}_{\text{v}}$$

where *M* is total body mass, *g* is gravitational acceleration (9.81 m·s^−2^), *h* is height of the body centre of mass, *V*_ap_ and *V*_v_ are the antero-posterior and vertical components of the linear velocities of the body centre of mass. W_ext_ is computed by summing the positive increments in ECM over the same period as for the determination of W_int_. W_tot_ is obtained from the arithmetic sum of W_int_ and W_ext_ by applying an approximation of König’s theorem of mechanics, which states that the total KE of a multilink system is made up of the KE of the segments relative to the overall centre of mass, which makes up W_int_, and the KE of the overall centre of mass, which is included in W_ext_ (Cavagna and Kaneko, 1977).

Mechanical work measures were expressed using the same units as energy cost (*Cr*; in J·kg^−b^·m^−1^) with *b* corresponding to 1, 0.75 (defined by literature) and 0.49 (determined allometrically). The conventional approach of considering only positive increments in mechanical energy (positive work) and neglecting negative work was applied. In level locomotion at a steady speed negative work is equal in magnitude but opposite in sign to positive work (and hence has no impact on comparison between groups). An inclusion of negative works would result in a network of zero which although mechanically correct is biologically meaningless and precludes determination of efficiency.

The Dvideo software (Laboratory of Biomechanics & Institute of Computing, UNICAMP; Campinas, Brazil) was used to calculate and track the bi-dimensional positions of the markers (see Figueroa et al., 2003) and, MATLAB software (Version 5.3, MathWorks, Inc.; Natick, Massachusetts, USA) was used in the reconstruction of 2D spatial model for both sagittal planes (filmed and estimated), signal processing and subsequent data over five strides. Anthropometric data of 11 rigid segments (head-trunk, upper arms, lower arms, thighs, shanks, feet) were used to compute the position of the segments and the body centre of mass. The low-pass Butterworth filter was used with automatic cut-off frequency selection for each marker. The range of cut-off frequencies was 8 to 11 Hz. Linear and angular velocity of each segment and linear velocity of body centre of mass was determined by numerical differentiation.

### Statistical analysis

The Shapiro-Wilk test was used to verify the normality of the data. The Pearson product-moment correlation test was used to analyze the relationship between mechanical work and long-distance running performance. Possible differences in VO_2max_ and mechanical work values, with and without the application of allometric exponents, were analyzed using one-way ANOVA with the Bonferroni Post-Hoc test based on the type of variance. Significance was accepted as *p* ≤ 0.05, statistical power observed 90%, and analyses were performed in SPSS 20.0. Results are reported as means ± *SD*.

## Results

**Figure 3:**
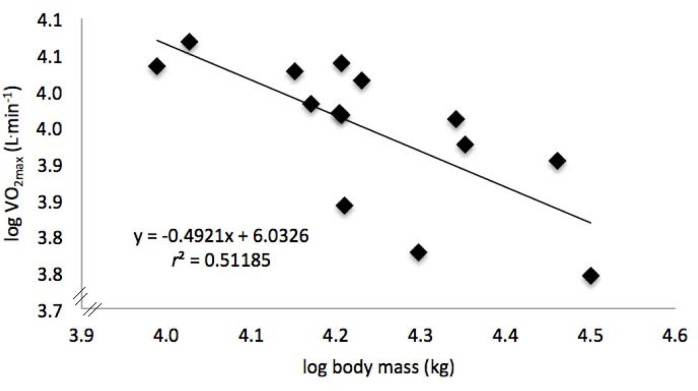
*Relationships between maximal oxygen uptake expressed in absolute terms (VO_2maxabs_) and body mass by using allometric log-linear for 14 recreational long-distance runners. Linear regression is shown with a 95% confidence interval.*

The relationship between VO_2max_ and body mass in recreational long-distance runners conformed to the allometric expression (Figure 3), and significant differences in VO_2max_ values with and without the application of allometric exponents were observed (Table 1).

Significant differences were observed in mechanical work, with and without the application of allometric exponents (Table 2). However, no significant correlations were found between mechanical work and VO_2max_.

Significant correlations were found between mechanical work and the performance of recreational long-distance runners, indicating that mechanical work could be a significant predictor of long-distance running performance in this population. However, the strongest correlations values were observed in W_ext_ and W_tot_, especially when using the specific allometric exponent (Table 3).

The W_ext_ showed higher values than the W_int_ (Δ = 51.6%), regardless of *b* values. Significant correlations were found between W_int_ and W_ext_ with W_tot_ for *b* = 1 (*r* = 0.86 and 0.81), *b* = 0.75 (*r* = 0.84 and 0.85) and *b* = 0.49 (*r* = 0.83 and 0.88).

## Discussion

The present study examined the effect of allometric scaling on the relationships between mechanical work and the performance of recreational long-distance runners. The main result of our study was the ability of the mechanical work to predict the performance in recreational long-distance runners, especially when the allometric exponents were applied.

The efficiency of locomotion is influenced not by the elastic energy storage relative to body mass, but by the elastic energy storage relative to the mechanical work of locomotion (Bullimore and Burn, 2005); an effect could be observed for example in Kenyan and Ethiopian runners that often dominate middle- and long-distance events in athletics.

For these subjects running is systematically more economical because they generally have a smaller body size and thinner lower limbs than other runners (Foster and Lucia, 2007), a result that demonstrates a probable relationship between mechanical work and running performance, and justifies our findings.

This important result can also be discussed in terms of the mechanical efficiency. According to Minetti et al. (1995) humans tend to choose a stride frequency that minimizes the W_int_ and W_ext_, which increases the mechanical efficiency and improves the running efficiency. The decrease in W_ext_ is mainly due to a reduction in vertical power, and W_int_ due to an increase in stride length. According to Willems et al. (1995) the W_int_ can be affected by external forces. The two effects considered are (i) equal and opposite vertical movements against gravity and (ii) the effect of the velocity changes of the body centre of mass. Our study also demonstrated an inverse relationship between mechanical work and performance in recreational long-distance runners, proving that the mechanical work and running efficiency are two important factors in long-distance runners performance.

Previous research indicates that in walking W_ext_.km^−1^ decreases with speed (Cavagna, 2010), while W_int_·km^−1^ increases (Cavagna and Kaneko, 1977). Furthermore, in running, W_int_ is lower than W_ext_, about 5.5 m·s^−1^, whereas at the highest speeds the reverse is true (Cavagna and Kaneko, 1977), demonstrating the importance of this mechanical variables in running performance. In our study W_ext_ showed higher values than W_int_ for 3.1 m·s^−1^ and strongest correlations with the performance in comparison to W_int_, corroborating with Cavagna and Kaneko (1977). In fact, in non-fatigued running, W_ext_, corresponding to the work necessary to lift and accelerate the body center of mass within the environment (Saibene and Minetti, 2003), has been significantly more important than W_int_, and related to inter-individual running efficiency differences in the literature (Candau et al., 1998).

The effective mechanical advantage (EMA = *r*/*R*, where *r* is the muscle mechanical advantage and *R* is the ground reaction force) for muscle force production also can contribute to the understanding of the relationship between body mass and mechanical work. In a study conducted by Biewener et al. (2004), an association between EMA was verified and kg^0.52^ of body mass in humans during running. A similar *b* value was found in our study. Biewener (1989) demonstrated that this relationship may be understood based on physiological processes and a size-dependent change in locomotor limb posture: small animals run with crouched postures, whereas larger species run more upright. By adopting an upright posture, large animals align their limbs more closely with the ground reaction force, substantially reducing the forces that their muscles must exert (proportional to body mass) and hence, the forces that their bones must resist, to counteract joint moments, resulting thus in a specific *b* value. According to this author, a greater energy cost during running in humans, is a consequence of a great W_int_ and W_ext_ (Minetti et al., 1994), what may be explained in part by a decrease in limb mechanical advantage.

The relationship between body mass and metabolic rate, especially the *b* value, has attracted the interest of biologists and healthcare professionals throughout the world (Ingham et al., 2008; Tartaruga et al., 2010). Our study verified a low specific allometric exponent in maximal metabolic rate (*b* = 0.49, Figure 3), demonstrating that the scaling behaviour of human metabolic rate is, to a great extent, dependent on the physiological state (Markovic et al., 2007). Jensen et al. (2001) showed a considerable variability of *b* values (0.19 to 0.92) in different sports, including running (*n* = 20; *b* = 0.59), triathlon (*n* = 16; *b* = 0.24) and walking (*n* = 6; *b* = 0.19). West et al. (1997) demonstrated that allometric scaling may be understood in terms of bases that limit supply and/or physiological processes that contribute to the regulation of a metabolic rate, as proposed later by Darveau et al. (2002). This demonstrates the existence of specific allometric exponents and is contrary to the theories initially proposed by Rubner (1883) (*b* = ⅔) and Kleiber (1947) (*b* = ¾). Scaling of a maximal metabolic rate has been related to aerobic capacity of humans and other animal species; whereas in animals and untrained humans the largest species of individuals have the greatest aerobic capacity, the opposite is true for human athletes, where the smallest endurance athletes exhibit the greatest aerobic performance (Markovic et al., 2007). Thus, it is not surprising that recreational runners or animals in general exhibit differential scaling behaviour of a maximal metabolic rate compared to human athletes. Furthermore, the negative relationship between muscular activation during aerobic exercise and strength measurements verified by Cadore et al. (2011) suggests that there is an interaction between running efficiency and mechanical work.

Physiologically, oxygen uptake at a given submaximal running velocity is not proportional to body mass; i.e., the oxygen uptake per kg of body mass displayed an inverse relationship to body mass (Bergh et al., 1991) and this is in agreement with data from animal studies (Taylor et al., 1982), as well as from experiments involving humans (Thorstensson, 1986). In one of the first studies that used an allometric exponent to express running efficiency, Bergh et al. (1991) found that oxygen consumption during running is better related using specific allometric exponents, for example, kg^−¾^ and kg^−⅔^, than to kg^−1^.

In mechanical terms, experimental evidence confirms that the contribution of elastic energy to the mechanical work of locomotion does not increase as rapidly with size as the mass-specific energy storage capacity, suggesting that the percentage contribution of elastic energy to the mechanical work of locomotion decreases with size. The reason for this is that the mechanical work of locomotion per kilogram of body mass is directly proportional to the distance travelled (Blickhan, 1989), so that subjects with larger body size, with their longer strides, must perform relatively more work per stride. Because each tendon can store and return elastic energy only once per stride, this greater mechanical work will tend to offset the greater elastic energy storage capacity of larger runners. Therefore, the contribution of elastic energy to the mechanical work of locomotion cannot increase with size as rapidly as the energy stored per stride, and could be greater in smaller runners, demonstrating that the relationship between body weight and mechanical parameters is not linear and suggesting the application of allometric models.

## Conclusion

Existing research indicates that oxygen consumption does not increase proportionally to body mass during running activities (Bergh et al., 1991; Brisswalter et al., 1996; Nevill et al., 2004; Foster and Lucia, 2007). As such, dividing oxygen uptake by body mass may produce erroneous interpretations when comparing individuals or groups who differ in body mass. In weight-supported events, studies have indicated that mechanical efficiency, dependent on mechanical work (in J·kg^−1^·m^−1^), is clearly an important predictor of endurance running performance. Studies have demonstrated that allometric scaling can improve the relationship between running efficiency and performance, but the relationship between mechanical work and performance has not yet been reported in scientific literature. This study revealed that mechanical work may predict recreational long-distance performance and an allometric model may improve this prediction, suggesting that the use of allometric scaling is limited according to the aerobic capacity or morphofunctional parameters of an individual. Strategies for improving or predicting endurance running performance are yet to be developed, although it appears that allometric scales may be a common element that improves this prediction for recreational long-distance runners.

## Practical Application

Differences in body mass account for almost 70% of the differences observed in VO_2max_ and running efficiency in recreational runners. Generally measured per unit mass basis, it reduces the obvious disparities that will be observed in runners of differing total body mass. However, while expressing VO_2max_ or running efficiency on a per unit weight basis will control for differences in total body mass, it does not eliminate the differences in body composition, demonstrates that an indiscriminate use of kg^−1^ is inappropriate to compare subjects with different body characteristics. The findings of the current study demonstrate that the allometric model is a good method to determine and compare the endurance performance prediction in recreational endurance runners; an alternative, simple and interesting method that can improve the effectiveness of aerobic training.

## Figures and Tables

**Table 1:** Running performance in 10000 m and physiological characteristics of 14 recreational long-distance runners^*^

Running performance (min:s)	43:38 ± 07:20
VO_2max_ (ml·kg^−1^·min^−1^)	52.0 ± 4.9
VO_2max_ (ml·kg^−0.75^·min^−1^)	149.8 ± 11.1
VO_2max_ (ml·kg^−0.49^·min^−1^)	446.6 ± 29.9
Heart rate _max_ (bpm)	189 ± 16

* Values are mean ± SD.

**Table 2:** *Mechanical work at 3.1 m*·s^−1^of 14 recreational long-distance runners^*^

	*b* = 1		*b* = 0.75		*b* = 0.49	

	Mean	*SD*	Mean	*SD*	Mean	*SD*
W_int_ (J·kg^−b^·m^−1^)	0.63	± 0.13	1.82	± 0.39	5.43	± 1.23
W_ext_ (J·kg^−b^·m^−1^)	1.22	± 0.13	3.52	± 0.45	10.53	± 1.62
W_tot_ (J·kg^−b^·m^−1^)	1.85	± 0.22	5.33	± 0.71	15.96	± 2.46

*Internal Mechanical Work (W_int_); External Mechanical Work (W_ext_); Total Mechanical Work (W_tot_). Allometric exponent (b).*

* Values are mean ± SD

**Table 3:** *Relationships between mechanical works and running performance at 3.1 m*·*s^−1^of 14 recreational long-distance runners*

	*r*	Equation
W_int_ (J·kg^−1^·m^−1^)	0.38	*y* = 1240.3*W_int_* + 1821.5
W_ext_ (J·kg^−1^·m^−1^)	0.80	*y* = 2649*W_ext_* − 621.28
W_tot_ (J·kg^−1^·m^−1^)	0.70	*y* = 1356.3*W_tot_* + 98.662
W_int_ (J·kg^−0.75^·m^−1^)	0.44	*y* = 492.07*W_int_* + 1707.8
W_ext_ (J·kg^−0.75^·m^−1^)	0.83	*y* = 814.16*W_ext_* − 262.39
W_tot_ (J·kg^−0.75^·m^−1^)	0.77	*y* = 471.62 *W_tot_* + 86.151
W_int_ (J·kg^−0.49^·m^−1^)	0.50	*y* = 175.48*W_int_* + 1648.5
W_ext_ (J·kg^−0.49^·m^−1^)	0.82	*y* = 220.88*W_ext_* + 275.38
W_tot_ (J·kg^−0.49^·m^−1^)	0.78	*y* = 139.16 *W_tot_* + 380.36

*Internal (W_int_), external (W_ext_) and total (W_tot_) mechanical work. Running Performance in 10000-m (y). p = 0.05*
